# Psychodynamic Psychiatry Education and Training for Doctors

**DOI:** 10.1192/bjo.2022.124

**Published:** 2022-06-20

**Authors:** James FitzGerald, Fraser Arends, Pamela Peters, David Christmas

**Affiliations:** 1 Cambridgeshire and Peterborough NHS Foundation Trust, Cambridge, United Kingdom.; 2 University of Cambridge, Cambridge, United Kingdom

## Abstract

**Aims:**

Background and Aim: Psychodynamic psychiatry training seminars are a blended supervision and experiential style approach to training health care professionals in reflective practice and formulation. They apply psychodynamic theory through case formulations, seminars, and Balint groups so that health care staff can improve their communication style, formulation skills and enhance their appreciation for patients with complex mental health problems. Our aim is to evaluate the provision of our psychodynamic psychiatry training sessions for doctors in psychiatry, gastroenterology, and emergency medicine, and to evaluate the perceived benefits of attending in terms of personal and professional development.

**Methods:**

Methods: The evaluation used a standardized mixed-methods approach, with the sample consisting of psychiatry core trainees (n = 9), gastroenterology higher trainees (n = 4), and emergency medicine doctors (n = 10). The evaluation period was between October 2021 and January 2022. Data were gathered via a survey tool, adapted from the literature using Likert scales and free text questions to identify barriers and facilitators to the sessions.

**Results:**

Results: All participants (n = 23) scored the group highly across the board in terms of acceptability, clinical impact, and fidelity measures. All participants reported that they have a better appreciation of group dynamics, the impact of the doctor's humanity and personality on their clinical work, and the symbolic meaning of the patient's symptoms. Notably, approximately 60% reported that the sessions were relevant to their ongoing training needs and that 95% of participants felt the sessions were a safe place to express and process anxieties and frustrations about their work. All participants either agreed or strongly agreed the group had changed the way they think and practice, and that they felt able to consider their clinical encounters in a new light.

**Conclusion:**

Conclusion: This evaluation reports early findings on psychodynamic psychiatry teaching for different medical groups. Overall, the participants felt the sessions were relevant to their training and improved their personal and professional development. Key benefits of the group were highlighted and included increased insight into the emotional and symbolic aspects of the patient's symptoms and clinical issues, team working through cohesion, and the humanity of the doctor in the clinical relationship with the patient. This suggests that the sessions provide a much-needed space to process and reflect on the often-intense demands of clinical work, individually and as a team. The main theme within barriers to the group processes was external in terms of other clinical demands requiring prioritization.

